# Propagation of Parkinson's disease by extracellular vesicle production and secretion

**DOI:** 10.1042/BST20220204

**Published:** 2022-09-16

**Authors:** Laura E. Shippey, Susan G. Campbell, Andrew F. Hill, David P. Smith

**Affiliations:** 1 Department of Biosciences and Chemistry, Sheffield Hallam University, Sheffield, U.K.; 2 Department of Biochemistry and Chemistry, La Trobe Institute for Molecular Science, La Trobe University, Bundoora, Australia; 3 Institute for Health and Sport, Victoria University, Footscray, Australia

**Keywords:** α-synuclein, extracellular vesicles, Parkinson's disease

## Abstract

Parkinson's disease (PD) is a common neurodegenerative condition affecting a significant number of individuals globally, resulting in the presentation of debilitating motor and non-motor symptoms, including bradykinesia, resting tremor, as well as mood and sleep disorders. The pathology of PD has been observed to spread through the central nervous system resulting in progressive brain degeneration and a poor prognosis. Aggregated forms of the protein α-synuclein, particularly intermediary aggregates, referred to as oligomers, or preformed fibrils, have been implicated as the causative agent in the degeneration of neuronal processes, including the dysfunction of axonal transport, mitochondrial activity, and ultimately cellular death. Extracellular vesicles (EVs) have been strongly implicated in the propagation of PD pathology. Current observations suggest that aggregated α-synuclein is transported between neurons via small EVs in a series of exocytosis and endocytosis cellular processes leading to the observed spread of neurotoxicity and cellular death. Despite some understanding of the role of EVs in neurodegeneration, the exact mechanism by which these lipidic particles participate in the progression of Parkinson's pathology is not entirely understood. Here we review the current understanding of the role of EVs in the propagation of PD and explore their potential as a therapeutic target.

## Introduction

Parkinson's disease (PD) is the second most common neurodegenerative disease after Alzheimer's disease (AD), with a reported age-standardised incidence rate of 13.43 per 100 000 individuals in 2019 [1]. The disease is characterised by the loss of nigrostriatal dopaminergic neurons and non-dopaminergic neurons [2]. Clinically, PD is described as a heterogenous condition [3] with motor symptoms often consisting of akinesia, bradykinesia, rigidity, tremor, and changes of gait [4]. Patients also exhibit non-motor symptoms such as pain, sleep dysfunction, and psychiatric disorders [5,6]. Environmental risk factors that bring about cellular stress have also been linked to the progression of PD, including brain traumas and exposure to pesticides [7]. Currently, there are no disease-modifying treatments or neuroprotective therapeutics available for patients diagnosed with PD. Levodopa, a dopamine precursor pharmacological agent, remains the most effective therapy [8,9]. However, long-term usage of this drug can result in various forms of dyskinesia [10], hallucinations, and other adverse effects [11]. A central hallmark of the disease state is the presence of Lewy bodies in surviving neurons. These are composed primarily of a misfolded form of the protein α-synuclein aggregated into amyloid structures which has been associated with compromised cellular activity and eventual cell death. α-Synuclein within these inclusions is phosphorylated at serine-129 (S129), with this post-translational modification acting as a marker for neurodegeneration [12]. These cells also show signs of extensive oxidative and endoplasmic reticulum-related stress [13]. The mechanism by which α-synuclein is induced to aggregate within the cell and the pathological spread between cells in the diseased brain is not fully understood. Extracellular vesicles (EVs) along with factors such as *SNCA* genetic mutations, aggregation of α-synuclein, and cellular stresses have been implicated in the propagation of PD and are thought to play a central role in pathology [14–18]. In this review, we will explore the role of EVs in propagating PD pathology.

## Establishing the connection between extracellular vesicles and α-synuclein in PD pathology

### The cellular role of extracellular vesicles

EVs are membranous vesicular structures containing a range of biomolecular cargo secreted into the extracellular space by cells [19]. The ability to transfer biological molecules between cells has implicated EVs in numerous diseases such as cancer [20], cardiovascular diseases [21], and neurological disorders [17]. Various EV subtypes include exosomes, microvesicles (MVs), and apoptotic bodies [22]. Exosomes are nano-sized structures, ranging from 30 to 150 nm in diameter and are formed from the invagination of the late endosomal membranes within limited multivesicular bodies (MVBs). These MVBs fuse with the cellular plasma membrane releasing intraluminal vesicles into the extracellular space [23,24]. MVs, are more heterogeneous in their size span, primarily observed to be up to 1000 nm in diameter. MVs are produced and released into the extracellular space through plasma membrane outward budding and pinching, also called ‘shedding’ [23,25] or ectocytosis [26]. EVs are specialised, dynamic structures produced by a wide range of cellular types with essential roles in endocytosis and cellular communication [27,28]. The biomolecular cargo carried by these EVs varies upon the cell type they are released from [29]. Typically, exosomes and MVs have been found to contain cytosolic, membrane and nuclear proteins, lipids, messenger RNA (mRNA), micro-RNA (miRNA), and non-coding RNA species [30]. Despite its physiological importance, EVs have been implicated in PD and other neurodegenerative diseases [31], with exosomes and MVs primarily associated with neuropathology [17]. In the context of PD, α-synuclein has been shown to co-localise with cellular endocytic components such as endosomes and lysosomes [32]. Further, EVs have been observed to contain misfolded α-synuclein aggregates at the cell body and the neuronal synapse for the purpose of release into the extracellular space [33]. EV-mediated transfer of α-synuclein has been shown to occur between neurons as well as between neuronal and glial cells such as microglia and astrocytes, potentially contributing to PD pathology [34–36].

### The role of α-synuclein in PD

At the core of PD pathology, is α-synuclein, a 140-residue protein encoded by the *SNCA* gene. The protein has three identified domains, a lipid binding N-terminal region, the central non-amyloid component (NAC), and the negatively charged C-terminal region. The protein is primarily localised in the presynaptic nerve terminals and can bind to SNARE complexes through interactions with synaptobrevin-2/VAMP-2 [37], regulating neurotransmitter release [38] as well as having potential roles in EV maintenance and rearrangement [39]. Although this protein has a physiological role in normal neuronal activity, it has been found to play an essential role in PD pathology [40–43]. Wild-type (WT) and mutated forms of α-synuclein will misfold and aggregate into a range of oligomeric species, protofibrils, and mature amyloid fibrils [44,45]. These amyloid aggregates form integral components of Lewy bodies in the cell soma and Lewy neurites in the axons of surviving neurons [45–48]. The familial mutations such as A30P, A53T, E46K, H50Q, G51D, and A53E which are localised in the N-terminal region, have been found to alter the association of α-synuclein with lipid membranes and contribute to changes in the aggregation processes [49]. Studies that have previously compared the toxicity of monomeric, oligomeric, and fibrillar forms of α-synuclein showed oligomeric conformations displayed greater toxicity to cell lines than fully formed fibrils and monomers implicating them as an important pathological agent in PD [50–52]. These oligomeric aggregates are able to induce cell death [53] through compromising cellular proteasome activity [54], impeding mitochondrial respiration [55], inducing cell membrane permeabilization [56], causing oxidative [57], and/or endoplasmic reticulum stress [58] and seeding intracellular aggregation of endogenous α-synuclein [59]. This ability to bring about intracellular aggregation imparts a prion-like mechanism involving the oligomeric forms of α-synuclein. As such, the spread of oligomeric forms of α-synuclein between cells is thought to play a central role in the pathology of the disease. Specific fibrillar aggregates known as preformed fibrils (PFFs) have been highlighted as neurotoxic and are assumed to form in neuronal cells due to amyloid fibril fragmentation [60–62]. Fragmentation is believed to be caused by chaperone proteins, lysosomal proteases, and endosomes/lysosomes inducing acidic conditions [60–62]. Experimentally, PFFs are produced via the sonication of amyloid fibrils forming aggregates approximately 50 nm in length. These assemblies exhibit a propensity to recruit endogenous α-synuclein inducing the seeding of further aggregation in neurons in both cellular and animal models [63,64].

### The prion-like nature of α-synuclein in the context of PD

Prions are misfolded proteins that undergo propagation involving a structural transitional process from a native morphology to a misfolded conformation [65]. In humans, there are a range of rare disease states such as Creutzfeldt–Jakob disease (CJD) in which the native cellular prion protein, PrP^C^, undergoes a structural conversion from an α-helical structure to a β-sheet rich conformation. This transformation forms the toxic and proteasome clearance resistant PrP^Sc^ (scrapie isoform of the prion protein). PrP^Sc^ can further aggregate through the recruitment of PrP^C^ and the templated conversion to PrP^Sc^. Ultimately the presence of these aggregates induces rupturing of cells causing the shedding of PrP^Sc^ proteins. Studies have reported the presence of PrP^c^ and PrP^Sc^ in neuronally derived exosomes indicating the potential role of exosomes in cell-to-cell transmission and the propagation of prion disease pathology [66–68]. The existence of proteins that exhibit similar characteristics in structure and activity to that of prions are referred to as ‘prion-like’ proteins (PrLPs). PrLPs are thought to contribute to the pathology of neurodegenerative conditions such as AD, PD, and amyotrophic lateral sclerosis [3]. The concept of α-synuclein as a PrLP arises from insights in both *in vitro* and *in vivo* models. Studies have highlighted that exposure to aggregated α-synuclein in the form of oligomers or PFFs results in the propensity of endogenous α-synuclein to misfold and induce further aggregate formation in recipient cells [63,69]. Further, α-synuclein aggregates, with the ability to provide a template for further aggregation, have been detected in EVs, giving a potential mechanism by which the cell-to-cell propagation of PD pathology can be mediated [33,70].

## Factors influencing extracellular vesicle packaging and release in Parkinson's disease

### The role of SNCA mutations in extracellular vesicle packaging and processing

The connection between EVs and PD pathology has become more established [16]. However, a full understanding of the processes and conditions that allows EVs to mediate the spread of pathology is unclear and are required for a full understanding of neurodegeneration in PD. Mutations in the *SNCA* gene have been associated with increased α-synuclein aggregation and early onset PD [71,72]. Interestingly, these mutant forms of α-synuclein have also been shown to impact the packaging of this protein into EVs. Enzyme-linked immunosorbent assay showed that the A53T mutation, when present in SH-SY5Y neuroblastoma cells was more likely to show an increased association of α-synuclein to EVs. This is thought to be due to the increased rate at which the fibrillar aggregates are formed [16]. Which supports the concept that increased aggregation alters the packaging of α-synuclein into EVs. Comparable results were identified in human iPSCs expressing aggregation-prone A53T and E46K mutations, where an increase in phosphorylated α-synuclein was also observed in EVs encapsulated in MVBs [73]. Further, when α-synuclein was incubated with exosomes derived from mouse neuroblastoma cells expressing the mutations A53T, E46K, or A30P there were subsequent observations of accelerated rates of aggregation into fibrils [74].

### The role of SUMOylation in extracellular vesicle packaging

In addition, to genetic mutations and increased aggregation, post-translational modifications of α-synuclein can be integral to its packaging in EVs. The organisation and packaging of α-synuclein into EVs have been documented to be influenced by SUMOylation, a process which also serves to act as a sorting signal for EV release [75]. This process involves proteins called SUMOs which are small-ubiquitin-like modifiers [76,77]. SUMOylation involves SUMO-specific proteases processing SUMO proteins ultimately allowing the SUMO-conjugating enzyme Ubc9 to bind to the consensus site, ψ-K-x-D/E. A mechanism which is important in DNA repair and organisation of nuclear components [75]. This process is sensitive to cellular stressors as seen when rotenone was injected into the brains of mice leading to an increase in SUMO1 expression coinciding with increased α-synuclein levels [78]. Similarly, in oxidative stress conditions, DJ-1, a protein implicated in PD that is key in preventing cell death through antioxidation, is modified by SUMOylation at its lysine-130 residue [79]. Evidence has shown α-synuclein undergoes SUMOylation in the presence of an E3 ligase and SUMO conjugation has been observed to occur between α-synuclein and SUMO2 in the brains of mice [80]. *In vitro* SUMOylation has been shown to prevent amyloid fibril formation and promote protein solubility [80]. However, α-synuclein mutants, A53T, A30P, and E46K which are prone to aggregation, are also more susceptible to PIAS2-dependent SUMOylation. Consequentially, this inhibits ubiquitination of α-synuclein aggregates resulting in their accumulation within the cell. The overexpression of PIAS2 also results in increased levels of α-synuclein extracellularly [77]. SUMOylation has been shown to result in increased levels of extracellular α-synuclein [77] plus EVs have been shown to contain SUMO2, which is dependent on the endosomal sorting complex required for transport [75]. Which suggests SUMOylation may be connected to extracellular release. When N2a cells were transfected with Myc-α-synuclein mutants which interferes with SUMOylation of the protein, EV fractions were found to contain less of the mutant proteins [75]. Additionally, use of a small interfering RNA (siRNA) to silence of Ubc9 reduced α-synuclein release via EVs [75]. Further, studies have indicated that SUMOylation may increase ubiquitination or compete at target lysine sites at which both SUMOylation and ubiquitination can occur [77,80]. It may be reasonable to consider that SUMOylation is a potential explanatory post-translational event for the altered packaging and release of α-synuclein in pathological stress conditions.

### The role of cellular stress in extracellular vesicle packaging

Cellular stress in general is known to influence EV production and cargo contents [81,82]. The release of EVs into the extracellular space is also governed by the cellular environment and cell stress, and has been shown to be influenced by adiponectin [83], leptin, radiation, inflammation, hyperglycaemia, and hypoxia [84]. Cellular stress may participate in neuropathology as patients diagnosed with PD have been shown to express high levels of cerebrospinal fluid stress markers compared to control participants, including ferritin, 8-OhdG, nitrite, and malondialdehyde [82]. Treatment of cells with thapsigargin, which induces endoplasmic reticulum stress and increases the cytosolic calcium concentration results in the increased secretion of α-synuclein via EVs [70]. Similarly, when cells were treated with the calcium ionophore ionomycin, there was an increase in α-synuclein transport to the extracellular space via exosomal vesicles [70]. This increased association of α-synuclein to EVs has been demonstrated to be linked to the increased levels of calcium which enhances the ability of α-synuclein to bind to lipid membranes via its C-terminus [85].

Like exosomes, MVs shedding/release into the extracellular space is also affected in the conditions of stress such as hypoxia, dysfunctional calcium ion homeostasis, and compromised actomyosin coordination [25]. Moreover, the release of exosomes and MVs are regulated by cellular stresses such as excess reactive oxygen species which consequently alters the type and amount of cargo carried within these vesicular subtypes [81]. This was demonstrated with mouse mast cells, where oxidative stress (induced by hydrogen peroxide treatment) caused alterations in exosomal mRNA [86]. In addition, the induction of oxidative stress in red blood cells with *tert*-Butyl hydroperoxide caused a subsequent increase in MV production [87]. In the context of PD, cortical neurons were found to secrete EVs containing both aggregates and phosphorylated S129 α-synuclein. When cortical neurons were treated with the lipid peroxidation product 4-hydroxynoneal, increased amounts of α-synuclein oligomers and fibrils were detected in EVs compared to EVs released from the control cortical neurons [88]. Therefore, external stressors may be necessary when assessing the potential causes of α-synuclein neuron-to-neuron propagation via EVs.

When α-synuclein accumulates or aggregates in the cell, it has been found to result in the inhibition of lysosomal function through the reduction in the activity of hydrolases [89]. Dysfunction of the lysosomal system has also been shown to be implicated in PD pathology [90,91]. The connection between the inhibition of the autophagy-lysosomal pathway (ALP) and higher levels of α-synuclein cargo in EVs has also been shown [92]. Endocytosis, of α-synuclein fibrils can induce lysosomal stress and induce autophagic responses that lead to the release of α-synuclein from the cell [93]. Furthermore, it has been found that the secretion of α-synuclein in the context of impeded lysosomal function is mediated by LGALS3, which codes for the protein galectin 3. Following treatment with lysosomal acidification inhibitors, Baf-A1, and Chloroquine, EVs were found to contain α-synuclein as well as galectin 3, a process that is increased when disturbance of the ALP occurs [93]. Treatment with α-synuclein fibrils resulted in observations of endogenously expressed SNCA and exogenous SNCA fibrils colocalising with galectin-positive intracellular vesicles and, ultimately, in EVs released from cells. Galectin 3 depletion, in the event of α-synuclein fibrillar treatment, was also shown to lead to autophagic impairment due to a reduction in the formation of autophagosomes [93]. Together this data suggests that LGALS3 (galectin 3) in conjunction with other proteins is important in the regulation of aspects of ALP, and for cellular secretion of α-synuclein.

## The implications associated with extracellular vesicle cargo internalisation in Parkinson's

### The processes involved in extracellular vesicle internalisation

The internalisation of neurotoxic aggregates via EVs and release of cargo into the recipient cells is integral to cell-to-cell transmission of α-synuclein induced PD pathology [94,95]. EVs can be taken into the cell through the endocytic processes, which encompasses but is not limited to clathrin-mediated endocytosis, caveolin-mediated endocytosis, macropinocytosis, lipid-rafts, and phagocytosis [96]. In addition, other internalisation processes involve cell surface membrane fusion, and cell-specific EV uptake through ligand–receptor interactions, and are discussed in the following review [96]. Following internalisation, the release of cargo has been shown to occur through EV fusion with the late endosome and lysosome which may indicate the mode of entry into the cell [97].

Cell surface proteins appear to be intrinsic in the internalisation process with receptors such as lymphocyte activation gene 3 (LAG3) and heparan sulphate proteoglycan (HSPG) mediating the endocytosis of aggregated α-synuclein [98]. Purified LAG3 D1 domain (L3D1) was found to preferentially bind to PFFs compared to monomeric α-synuclein as demonstrated using a bio-layer interferometry assay to measure binding affinity [18]. The exact molecular processes involved in such preferential binding are not completely understood however, L3D1 has been found to directly bind to the C-terminus of α-synuclein, which is known to be exposed in the aggregated fibrillar structure, potentially explaining LAG3's preferential association [18,99–101]. Deletion of the C-terminus prevents binding of monomeric α-synuclein and PFFs to L3D1 [18] indicating the C-terminal's importance in the cellular internalisation of α-synuclein. The phosphorylation of S129, a pathological indicator of Lewy bodies and Lewy neurites, has also been found to be important in the binding of fibrils to L3D1 [18,71]; S129E which mimics phosphorylated S129 increases the ability of α-synuclein, both monomer and PFFs, to bind to L3D1 [18].

The uptake of fibrils into the cell can also be guided by glycosaminoglycan (GAG) chains found on the cell surface which interact with amyloid structures via their positively charged regions [32]. Using B103 neuroblastoma cells, α-synuclein fibrils as opposed to oligomers were able to co-localize with the GAG heparan sulphate [32], expressed on cellular surfaces, the extracellular matrix and basement membrane [102]. This co-localisation has been demonstrated to occur in the endosomal/lysosomal pathway and is dependent on Rab5A (a GTPase protein) suggesting that heparan sulphate plays a key role in the cellular uptake of α-synuclein fibrils once exported [32]. Exposure of SH-SY5Y neuronal and KH1C oligodendrocytic cell lines to extracellular oligomeric forms α-synuclein results in the formation of large intracellular inclusions and small aggregates positive for α-synuclein when compared to control cells in a clathrin-dependent manner [103]. Dynamin 1 is one of the main dynamin proteins involved in clathrin-mediated endocytosis. It is expressed extensively on dopaminergic neurons, whereas dynamin 2, also involved in clathrin-mediated endocytosis, can be found on all cell types. Sertraline, a selective serotonin reuptake inhibitor (SSRI), can inhibit dynamin GTPase activity and prevent the uptake of α-synuclein. Further, the addition of sertraline to the cell culture medium prevents α-synuclein transmission and uptake between co-cultured SH-SY5Y (donor) and PC12 co-cultured neuronal cells (recipient) [103] which indicates that clathrin-mediated endocytosis is involved in α-synuclein cellular uptake.

### The role of post-translational modifications in EV internalisation of α-synuclein in Parkinson's disease

In addition to cell surface receptors, the internalisation process of α-synuclein aggregates is affected by post-translational modifications. O-linked *N*-acetyleglucosamine (O-GlcNAc), a post-translational modification in the form of protein glycosylation where *N*-acetyleglucosamine are attached to serine and threonine hydroxyl groups. This process is regulated by O-GlcNAcase (OGA), an enzyme intrinsic in removing this modification. When Thiamet-G was utilised to inhibit OGA, after 96 h, α-synuclein PFFs uptake into SK-N-SH cells was reduced by 35% [104]. This indicates increasing the levels of O-GlcNAc protein glycosylation post-translational modification prevents uptake of α-synuclein PFFs into SK-N-SH cells [104].

Phosphorylation is believed to be crucial in the neuropathological characteristics exhibited by α-synuclein. Aggregated α-synuclein has been shown to have extensive phosphorylation at S129 [47,105] and more recently discoveries of phosphorylation at tyrosine-39 have been found to alter the morphology of amyloid fibrils, specifically increasing the size of the fibril core, in rat cortical neurons contributing to neuropathology through its ability to seed fibrillization of WT α-synuclein [106]. However, S129 phosphorylation of α-synuclein has been more frequently observed in LBs in PD pathology and it is believed this post-translational modification heightens α-synuclein's ability to form β-sheet structures [107]. Further, S129 phosphorylation has been found to increase the ability of the A30P mutant α-synuclein to bind to lipid membranes which may be an important mechanism in the EV-mediated transport of α-synuclein between neurons [107]. Additionally, it has also been demonstrated in primary-culture mouse cells and mouse brains that exposure of cells to exogenous fibrils induced abnormal α-synuclein phosphorylation allowing for retrograde axonal transport of α-synuclein as well as subsequent aggregation in the cell body [108].

### The cellular implications of extracellular vesicle-associated internalisation of α-synuclein aggregates

**Figure 1.**
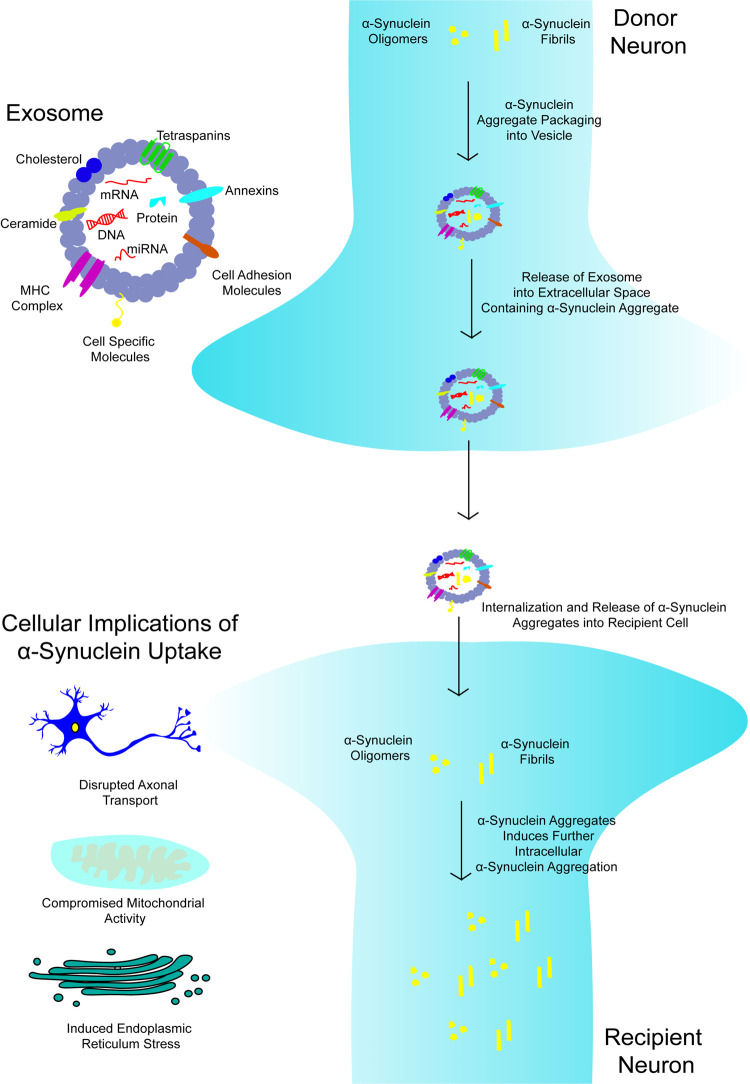
Extracellular vesicles mediate the transfer of aggregated α-synuclein. α-Synuclein aggregates present in the donor neuron interacts with the lipid membranes of EVs which allows its packaging and subsequent release into the extracellular space. Once in the extracellular space, α-synuclein is internalised into the recipient neuronal cells via EVs causing α-synuclein aggregate formation in the recipient neuronal cell due to its prion-like nature. The increased presence of neurotoxic aggregate species induces ER stress, compromised axonal transport, and mitochondrial activity dysfunction.

The uptake of EVs containing α-synuclein aggregates induces dysfunction in recipient neurons, including axonal transport dysfunction, hindering mitochondrial transportation, and disrupting the mitochondria's overall activity (Figure 1) [109]. Disruption of the mitochondria is seen to occur following cellular exposure to exogenous α-synuclein resulting in mislocalisation of the protein to the mitochondria and compromised cell viability [109]. Treatment of cells with exogenous α-synuclein has been demonstrated to impair mitochondrial membrane potential (MMP), increase the levels of mitochondria reactive oxygen species, and diminish ATP levels through the downregulation and silencing of Parkin as observed in PC12 neuronal cell lines [110]. Lentiviral infection was used to introduce either WT, E46K or E57K human α-synuclein into control iPSC-derived neurons. Levels of α-synuclein were found to increase in cells containing the mutant α-synuclein compared to cells expressing WT human α-synuclein. Size exclusion chromatography showed an increased presence of oligomeric aggregates in α-synuclein mutant cells [111]. Consequently, α-synuclein oligomers were found to reduce anterograde transport, leading to observations of clustered mitochondria leading to a reduction in ATP levels within the cells [111]. Neurons expressing mutant α-synuclein were treated with NPT-100-18A, a cyclic peptidomimetic compound that targets the C-terminus of α-synuclein. NPT-100-18A functions in displacing a-synuclein from the cell membrane, reducing aggregate formation [112]. NPT-100-18A treatment was also found to ameliorate the neurotoxic effects of the α-synuclein oligomers which was concurrent with the observation of increased mitochondria within axons and increased anterograde cellular transport [111].

Exosome-associated oligomers have also been shown to be associated with an increase in caspase3/7 activity, indicative of apoptotic cellular events, compared to exosome-free α-synuclein [33]. It is assumed that oligomers are encapsulated during the invagination process and then released in exosomes potentially due to the cell perceiving them as toxic, releasing the aggregated protein for self-preservation [33]. This notion was supported when autophagy-related gene 5 (ATG5), which is essential in autophagosome formation, was silenced in LUHME cells overexpressing α-synuclein, resulting in the increased release of α-synuclein into the extracellular space via exosomes. It appears the mechanism of secreting α-synuclein into the extracellular space is an attempt to reduce cell toxicity associated with α-synuclein accumulation caused by the failure of macroautophagy [113]. Altogether, the internalisation of α-synuclein via EV transport may act as a pre-requisite pathological event that induces α-synuclein seeding and subsequent neuronal dysfunction.

## Future directions and concluding thoughts

### Further research into EVs as a therapeutic target and biomarker in Parkinson's and neurodegeneration

Given the potential central role of EVs in the transmission of PrLPs, they have become an attractive target for pharmacological therapeutics. Pramiprexole is a pharmacological agent, that stimulates dopamine receptors and is assumed to have neuroprotective abilities through a reduction in capase-3 activity [114]. Exosomes isolated from the serum of patients diagnosed with PD who underwent treatment with pramipexole showed a decrease of α-synuclein in serum exosomes [115]. Additionally, when identifying exosomes in conditions of PD compared to multiple system atrophy (MSA), α-synuclein levels in EVs released from neurons were higher than that in EVs released from oligodendrocytes in patients diagnosed with PD. Whereas, in patients diagnosed with MSA, levels of α-synuclein are higher in exosomes released from oligodendrocytes compared to neurons [116]. The accumulating research surrounding the role of EVs in PD pathology presents them as a valuable therapeutic target and biomarker in distinguishing neurodegenerative conditions during patient diagnoses.

### Concluding thoughts

Current thinking suggests that small EVs are intrinsic to the propagation of the cellular pathology observed in PD. However, numerous factors such as *SNCA* mutations, post-translational modifications and cell stressors are essential in controlling the processing, packaging, and release of aggregated pathological α-synuclein. Furthermore, EVs themselves may pose as sites for the acceleration of α-synuclein aggregation, and successful uptake of aggregated neurotoxic α-synuclein. The consequences of exogenous a-synuclein aggregates uptake via EVs may be particularly detrimental to the functioning of the neuron, particularly pertaining to axonal transport and mitochondrial activity, potentially forming the basis for explaining the progressive cellular death observed in PD.

## Perspectives

- PD is associated with the misfolding and aggregation of the protein alpha-synuclein. EVs have been shown to play a role in the transfer of misfolded α-synuclein between cells and potentially mediate neuronal dysfunction.
- Targetting the loading of misfolded α-synuclein into EVs may be a potential therapeutic approach for PD and also offer potential diagnostic insights into this neurodegenerative disease.
- The mechanisms by which misfolded α-synuclein is associated with EVs and contribute to disease pathology are similar to other proteins which misfold and are associated with other neurodegenerative disorders such as Alzheimer's and prion diseases.

## Abbreviations

AD: Alzheimer's disease
ALP: autophagy-lysosomal pathway
EVs: extracellular vesicles
GAG: glycosaminoglycan
LAG3: lymphocyte activation gene 3
MSA: multiple system atrophy
MVBs: multivesicular bodies
MVs: microvesicles
OGA: O-GlcNAcase
O-GlcNAc: O-linked *N*-acetyleglucosamine
PD: Parkinson's disease
PFFs: preformed fibrils
PrLPs: prion-like’ proteins
WT: wild-type
